# Brucella Septic Arthritis: Case Reports and Review of the Literature

**DOI:** 10.1155/2016/4687840

**Published:** 2016-04-21

**Authors:** Fatehi Elnour Elzein, Nisreen Sherbeeni

**Affiliations:** Division of Infectious Diseases, Department of Medicine, Prince Sultan Military Medical City (PSMMC), Riyadh 11159, Saudi Arabia

## Abstract

Brucellosis is one of the commonest zoonotic infections worldwide. The disease is endemic in Saudi Arabia, the Middle East, and the Mediterranean area. Osteoarticular involvement is a frequent manifestation of brucellosis. It tends to involve the sacroiliac joints more commonly; however, spondylitis and peripheral arthritis are increasingly reported. Brucellosis can be overlooked especially in the presence of companion bacteria. Hence, it should be suspected in all patients with septic arthritis in endemic areas or in patients visiting such areas.

## 1. Case Reports

**Figure 1:**
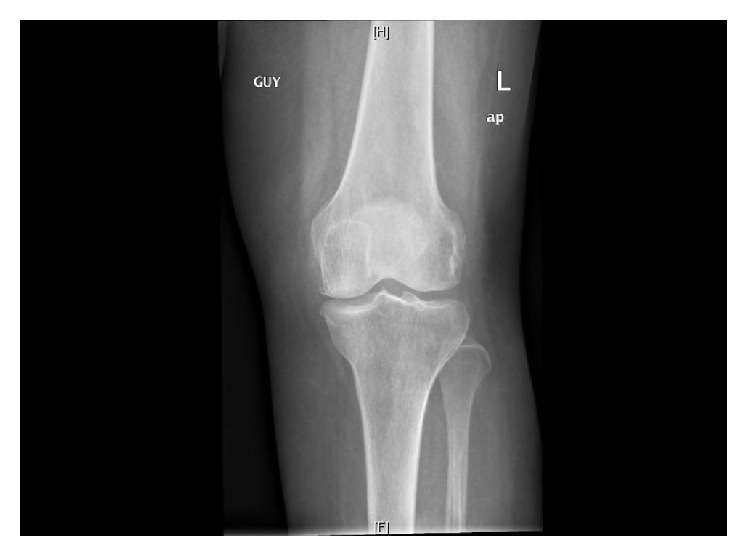
Moderate (L) knee joint effusion with OA.

*Patient 1*. A 72-year-old woman was admitted to hospital with (R) knee pain and swelling for 6/52. There are accompanying fever, night sweats, anorexia, and generalized body aches. She is known to have hypothyroid with osteoarthritis (OA) and polycythemia rubra vera. There was a history of raw milk ingestion. Her temperature was 38°C. The (R) knee was swollen, hot, and tender. General examination was normal except for splenomegaly. The WBC was 2.1 × 10^9^ and platelets were 618 × 10^9^ (150–450 × 10^9^). CRP and uric acid were high at 55 mg/L (<5 mg/L) and 624 *μ*mol/L (NR 143–339 *μ*mol/L) consecutively. The differential diagnosis included septic arthritis and crystal arthropathy. X-ray showed moderate left knee osteoarthritis with mild effusion (Figure 1). Synovial fluid aspirate was exudative with protein 34 g/L and high LDH 1036 but was unsuitable for cell count. An initially negative brucella serology, 2 months ago, is now positive 1 : 10240 (Table 1). Blood culture × 6 was positive; however, echocardiogram did not reveal vegetations. After 3-month treatment of rifampicin and doxycycline her symptoms subsided and CRP is 12.

**Figure 2:**
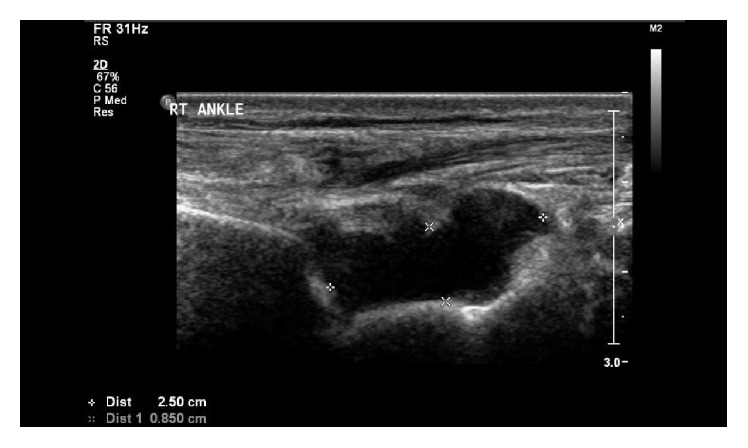
US of (R) ankle showing moderate effusion (3.8 cc).

**Figure 3:**
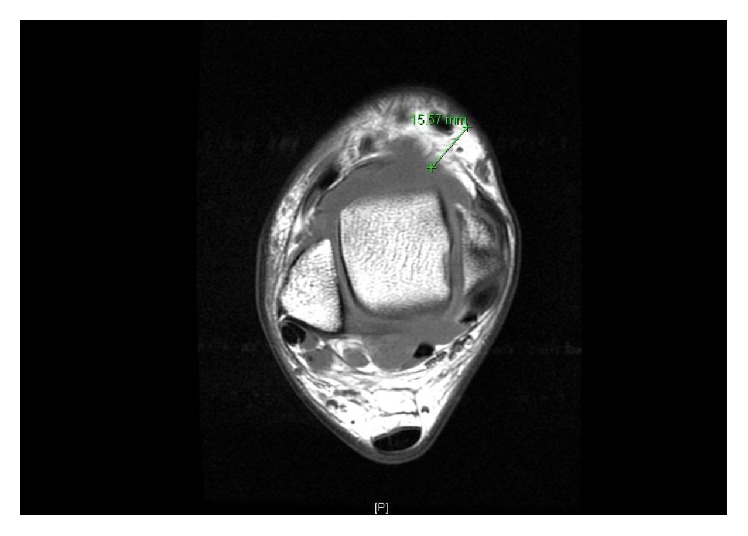
MRI showing a large (R) ankle effusion with tibialis posterior tenosynovitis.

*Patient 2*. A 21-year-old student presented with fever, drenching sweats, headache, generalized fatigue, and joints pain for three weeks. The general examination was normal except for fever and (R) ankle swelling. The joint was hot and tender with restricted range of movement. US and MRI of the (R) ankle showed large effusion with tibialis posterior tenosynovitis (Figures 2 and 3). His WBC was 7.7 × 10^9^, ESR 45 mm/hr., and CRP 53 mg/L. Synovial fluid was cloudy straw colored. The cell count was 3.3 × 10^9^ with 53% polymorphs. Brucella serology done by immunocapture assay was significantly raised 1 : 10240. Blood culture isolated* Brucella* species but synovial fluid culture was negative. He, his father, and his brother developed brucellosis concurrently. They all consumed the same raw milk. He received two weeks of streptomycin and a total of three-month rifampicin and doxycycline with full recovery. Follow-up CRP was 2 and ESR 1. 

**Figure 4:**
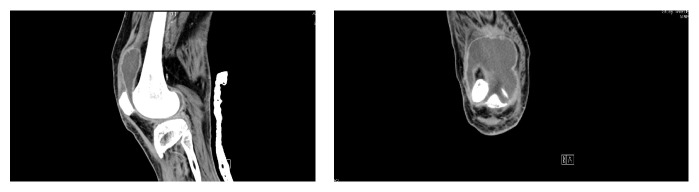
CT scan with moderate to large joint effusion of the (R) knee with associated synovial thickening and OA.

*Patient 3*. A 40-year-old diabetic patient was admitted with severe cellulitis of the feet after walking bare-footed on the sand. His diabetes is poorly controlled with HBA1C 15.5 (mmol/mol). His course was complicated by neuropathy and retinopathy. He also admitted to (R) knee swelling of two years duration. Multiple aspirations and injections of the knee resulted in temporary relief. There was no history of fever or night sweats. He had contact with animals but did not consume raw milk. The (R) knee was swollen, hot, and tender with restricted range of movement. Admission WBC was 8.7, CRP 38 mg/mL, albumin 24 g/dL, and alkaline phosphatase 220 *μ*/L. CT scan showed moderate to large effusion of the right knee with associated synovial thickening and osteoarthritic changes (Figure 4). Synovial fluid showed WBC of 9.9 × 10^9^ with 50.5% polymorphs. AFB smears and PCR for tuberculosis were negative. Synovial cultures isolated* Brucella* species but blood culture was negative. Brucella serology in the blood was high; it was 1 : 10240 and dropped to 1 : 640 on treatment. He received two weeks of streptomycin and completed a 3-month course of rifampicin and doxycycline. 

*Patient 4*. A 68-year-old man presented with (R) knee swelling and back pain for 4 months. He was initially diagnosed as OA and spondylitis. He is known to have DM, HTN, and IHD with a permanent pacemaker. CT scan showed degenerative spinal canal stenosis at L4-L5 with mild degree of bilateral SI joints degeneration. He had received epidural injection of steroid and (R) knee injection with temporary improvement. There was no history of fever or contact with TB patients; however, there was strong history of animal contact. He received one month of antibiotics prior to presentation but without improvement. General examination was normal but the (R) knee was swollen, tender, and with decreased ROM (10–15). CT scan showed moderate to large effusion of the (R) knee with associated synovial thickening and OA changes (Figure 4). The WBC was normal, ESR 70 mm/hr., and CRP 47 mg/mL, and rheumatoid factor was negative. The blood urea and creatinine was raised at 16.8 mmol/L and 140 *μ*mol/L. Synovial fluid cultures were positive for* Brucella* species but the blood culture was negative. Blood serology for brucella was 1 : 10240. He received 3/12 of doxycycline rifampicin with full functional recovery.

## 2. Discussion

We here present a group of four patients with brucella septic arthritis. All the patients are males except for one. Their age ranges between 21 and 72 yrs. The knee joint is affected in 75% of the patients while the ankle is involved in one patient. History of raw milk ingestion or contact with animals was found in all cases. One patient is part of a family cluster of brucellosis. Isolated focal symptoms were noted in one patient while the rest presented with associated systemic symptoms including fever and drenching sweats. Notably the WBC is normal in all the cases with ESR ranging between 20 and 70 mm/hr. This is in contrast to the brisk leukocytosis usually seen in other bacterial septic arthritis. The CRP's highest value was 87 mg/L. Blood and/or synovial cultures were positive in all patients. Of note, the brucella serology was consistently positive and at high titers. A minimum of two drugs for three-month duration was prescribed to all patients.

Brucellosis is one of the commonest zoonotic infections worldwide [1]. The infection can be acquired through consumption of unpasteurized dairy products, direct contact with infected animals, or animal's products of conception. Rare cases of mother to child transmission have been reported in relation to brucellosis during pregnancy and breast feeding [2]. Eye splashes especially in veterinary services and accidental inhalation in laboratory worker can lead to an increased risk of brucella infection. This high infectivity potential through inhalation renders brucellosis as a potential biological warfare agent [3].

Infections with* B. melitensis* persist as a major public health problem in Mediterranean countries; in western, central, and southern Asia; and in parts of Africa and South and Central America. Incidence estimates of more than 100 cases per 100 000 person-years have been reported in Mediterranean rim and the Middle East including Iraq, Jordan, and Saudi Arabia [4, 5]. Despite the apparent decline of brucellosis in certain parts of Saudi Arabia, it still remains an endemic disease with the highest rates among those 40–49 years of age [6].

Patients who develop brucellosis in many cases manifest a wide spectrum of symptoms including high fever, arthralgia, malodorous sweat, and splenomegaly. In other cases, the onset can be insidious and/or the involvement of a specific organ predominates (focal brucellosis). Osteoarticular involvement is one of the most frequent symptoms of brucellosis. A variety of disorders have been reported including sacroiliitis, spondylitis, peripheral arthritis, osteomyelitis, and bursitis. Furthermore, tenosynovitis similar to that seen in our second patient was described as early as 1908 with tendons of the wrist and ankles being the most commonly involved [7]. In a recent meta-analysis, arthralgia was present in 65% of patients while arthritis affected 26% of the infected group. Overall, spondylitis and sacroiliitis were detected in 12–36% of adults [8]. Peripheral arthritis constituting 38.8% of brucella arthritis manifested either as a single large lower limb joint or as an asymmetric pauciarthritis [9]. Three of our patients (75%) had a knee joint infection. This is in agreement with previous studies, where the knee joint was the most commonly affected joint [10]. Contrary to spondylitis, both sacroiliitis and peripheral arthritis tend to be nondestructive and heal without sequelae [9].

The diagnosis of brucella arthritis can be challenging especially in nonendemic areas. Serology, usually with standard agglutination tests (SAT), is the mainstay of diagnosis in endemic areas [5]. Traditionally, blood cultures have unpredictable sensitivity ranging from 53% to 90% and require prolonged incubation. When the disease is confined to a single joint, blood cultures may be negative, so serology remains the basis of laboratory diagnosis. Synovial fluid culture may remain positive despite negative blood cultures. Majority of our patients' synovial cultures were positive while only one patient had a positive blood culture. This is frequently achieved with synovial fluid collection in blood culture systems. The introduction of automated system has resulted in improved blood culture sensitivity reaching up to 95% and in incubation time as short as 7 days [11].* B. melitensis* grew in 14 of 15 (93.3%) BACTEC cultures, 6 of 8 (75.0%) isolator cultures, and 4 of 7 (57.1%) conventional cultures [11]. Analysis of synovial fluid usually demonstrates an exudative process, with leukocyte counts ranging from several hundreds to several thousands [12]. In general, the white blood cell count in the synovial fluid does not exceed 15 × 10^9^/L with lymphocytic predominance [13, 14]. Furthermore, synovial fluid analysis helps to distinguish crystal arthropathy from infectious arthritis, although the two occasionally coexist [15]. Although these procedures have resulted in a shortened incubation time, there is still a need for a more quick and reliable analysis. PCR have shown high sensitivity and specificity that could allow rapid and more sensitive identification of* Brucella* genus at the species and at the biovar level, compared with traditional techniques. However, its use remains infrequent mainly due to standardization problems [16]. Recent studies showed MALDI-TOF MS to be a rapid and highly reliable technique for straightforward brucella identification, both from culture plates and directly from blood culture vials [17].

Because monotherapy is frequently characterized by high rates of relapse, a combination of two drugs is used. The risk for overall failure with monotherapy was more than twice (relative risk 2.56) that of combination therapy [18]. The standard treatment is a combination of intramuscular streptomycin (0.75–1 gm) once daily for 2-3 weeks accompanied with doxycycline 100 mg twice daily for six weeks. Triple therapy for a course greater than 12 weeks is advocated in complicated brucellosis. Both therapeutic failure (3.02, 1.03–8.80) and relapse (1.70, 1.19–2.44) were significantly more common with the shorter duration of less than six weeks. Inversely, there is a lower relapse rate in the aminoglycoside/doxycycline than the rifampicin/doxycycline combination particularly in osteoarticular disease [18]. Meta-analysis revealed treatment failure or relapse of 5–7% for doxycycline–streptomycin regimens and 11–17% for doxycycline–rifampin [5]. A possible explanation is the lowering of doxycycline blood level by the simultaneous rifampicin administration [4]. Both in vitro susceptibility studies and molecular detection methods failed to demonstrate resistance to rifampicin. Overall, this high failure rate is likely related to poor compliance or inadequate duration rather than resistance to rifampicin. Similarly, the minimum inhibitory concentrations for other agents with the exception of co-trimoxazole remain reassuringly low. Of note, in areas where both TB and brucellosis are endemic, avoidance of rifampicin will obviate the risk of induction of resistance in tuberculosis, especially when TB is overlooked as the true diagnosis.

## 3. Conclusion

Brucellosis remains a challenging problem in endemic areas. Its variable rheumatologic manifestations can mimic different types of arthritis. In nonendemic areas, detailed travel and contact histories of patients are essential in order to establish an early diagnosis.

Blood, synovial cultures, and serology for brucellosis should be done in such cases. Combination of antibiotics and prolonged course of treatment is essential to prevent failure or relapse of brucella septic arthritis.

## Figures and Tables

**Table 1:** Summary of microbiology, serology findings, and the treatment of the patients.

Patient number	Age in years	Sex	Joint involved	Blood culture	Synovial culture	Serology	Treatment	Treatment duration
1	72	Female	(R) knee native	Positive	Positive	**1/10240**	Doxy/rifampicin	3 months
2	21	Male	(R) ankle native	Positive	Negative	**1/1280**	Doxy/rifampicin/streptomycin	3 months
3	40	Male	(R) knee native	Negative	Positive	**1/10240**	Doxy/rifampicin/streptomycin	3 months
4	68	Male	(R) knee native	Negative	Positive	**1/10240**	Doxy/rifampicin	3 months
